# *Ex vivo* Expansion of Human Adult Pancreatic Cells with Properties of Distributed Stem Cells by Suppression of Asymmetric Cell Kinetics

**DOI:** 10.4172/2157-7633.1000149

**Published:** 2013-09-14

**Authors:** JF Paré, JL Sherley

**Affiliations:** 1 The Adult Stem Cell Technology Center, Boston, MA, USA; 2 Tufts Center for Regenerative and Developmental Biology, Department of Biology, Tufts University, USA

**Keywords:** Diabetes, Adult stem cell, Tissue stem cell, Asymmetric self-renewal, Purine, Xanthine, Xanthosine, SACK, Expansion, p53

## Abstract

Transplantation therapy for type I diabetes (T1D) might be improved if pancreatic stem cells were readily available for investigation. Unlike macroscopic islets, pancreatic tissue stem cells could more easily access the retroperitoneal pancreatic environment and thereby might achieve more effective pancreatic regeneration. Unfortunately, whether the adult pancreas actually contains renewing stem cells continues as a controversial issue in diabetes research. We evaluated a new method developed in our lab for expanding renewing distributed stem cells (DSCs) from adult tissues as a means to provide more evidence for adult pancreatic stem cells, and potentially advance their availability for future clinical investigation. The new method was designed to switch DSCs from asymmetric self-renewal to symmetric self-renewal, which promotes their exponential expansion in culture with reduced production of differentiated cells. Called suppression of asymmetric cell kinetics (SACK), the method uses natural purine metabolites to accomplish the self-renewal pattern shift. The SACK purine metabolites xanthine, xanthosine, and hypoxanthine were evaluated for promoting expansion of DSCs from the pancreas of adult human postmortem donors. Xanthine and xanthosine were effective for deriving both pooled and clonal populations of cells with properties indicative of human pancreatic DSCs. The expanded human cell strains had signature SACK agent-suppressible asymmetric cell kinetics, produced Ngn3+ bipotent precursors for α-cells and β-cells, and were non-tumorigenic in immunodeficient mice. Our findings support the existence of pancreatic DSCs in the adult human pancreas and indicate a potential path to increasing their availability for future clinical evaluation.

## Introduction

Distributed stem cells (DSCs) [1–3], which are responsible for homeostatic renewal of differentiated organ and tissue cells, are the ideal cell type for use in cell replacement therapies. The quintessential DSC replacement therapy is transplantation of hematopoietic stem cells (HSCs) to restore blood cell formation. Though often effective, HSC transplantation therapy is still limited by an inadequate supply of HSCs. The sources of donor HSCs can be either autologous or allogeneic, but there are no means available to routinely grow larger numbers of HSCs from limited donor specimens [4].

While the challenge of DSC production only limits HSC therapy, it completely precludes cell therapy for most other types of organs and tissues. Though several other DSC transplantation therapies are being pursued (*e.g*., retinal, olfactory, and muscle stem cells) [5–9], they are all hampered by insufficient quantities of tissue-specific DSCs for transplantation. This situation has been a major driving force for current efforts to develop tissue-specific transplantation therapies based on human embryonic stem cells (hESCs) and induced pluripotent stem cells (iPSCs).

Once lines of these cells are established, they can be produced in large numbers. However, the understated wrinkle is that these pluripotent cells are improbable candidates for regenerative medicine, because they acquire many genetic and epigenetic defects; directing their production of mature tissue-specific functional cells is a formidable challenge; and they are prone to tumor formation [10].

The direct approach of expanding DSCs for regenerative medicine has been largely abandoned, because of the seeming impossibility of ever routinely propagating these cells in culture. We introduced the concept of suppression of asymmetric cell kinetics (SACK) as a new framework for understanding why DSCs were difficult to expand *ex vivo*. The key innovation in our thinking was recognition that a significant barrier to expansion of DSCs in culture was their characteristic asymmetric cell kinetics, which underpins their ability to maintain tissue cell homeostasis *in vivo* [11–17].

In the SACK method, cell culture media are supplemented with specific guanine ribonucleotide (rGNP) salvage precursors. These “SACK agents” allow DSCs to maintain high rGNP pool levels despite p53-dependent regulation of type II inosine 5′-monophosphate dehydrogenase (EC 1.2.1.14; IMPDH II), the rate-limiting enzyme for rGNP biosynthesis [18,19]. The purine compounds xanthosine (Xs) and xanthine (Xn) are effective SACK agents for the expansion of adult DSC populations originating from diverse mammalian species and tissues [14,16,17,20–23]. In this study, we adapted the SACK method for the *ex vivo* expansion of human adult pancreatic DSCs, which have potential for treatment of type 1 diabetes (T1D).

T1D is a debilitating disease resulting from destruction of the insulin-secreting β-cells in the pancreatic islets of Langerhans. T1D patients are unable to utilize glucose effectively, resulting in chronic hyperglycemia and its disabling sequelae. Current T1D treatment involves a combination of close monitoring of blood glucose and injection of insulin to control hyperglycemia. However, even with controlled pump technology, treatment regimens pale in comparison to the exquisite physiological blood glucose control by normal pancreatic islets. As a result, T1D patients succumb to multiple medical complications that result from a lifetime of inadequate glucose utilization control. Thus, a definitive cure requires restoration of normal islet function, which might be achieved by an effective pancreatic DSC transplantation therapy.

Transplantation of cadaveric islets of Langerhans has been approved for T1D treatment, but this source of pancreatic cell function is still inadequate [24]. An alternative approach would be transplantation of undifferentiated pancreatic stem cells that renewed pancreatic islet cell function *in vivo*. The identification of such cells within the human pancreas is a long and continuing controversy, and the candidates identified so far have shown limited capacity to proliferate *ex vivo*, a shortcoming that needs to be overcome in order to obtain sufficient stem cells to restore and renew therapeutic islet cell mass [25–27]. Herein, we report the results of our initial evaluation to determine whether the SACK method has potential for this purpose. Our findings show that this approach identifies a not previously described type of human adult pancreatic cells with stem cell properties that could enable new cell transplantation therapies for T1D.

## Materials and Methods

### Ethics statement

Postmortem donor human pancreas specimens certified for informed consent by referring institutions (http://ndriresource.org/Referral-and-Recovery-Process/43/) were obtained from the National Disease Research Interchange (NDRI). All studies with human donor specimens were reviewed and approved by the Boston Biomedical Research Institute Human Research Subjects Protection Committee.

### Cell isolation, SACK-expansion, and cloning

**Figure 1:**
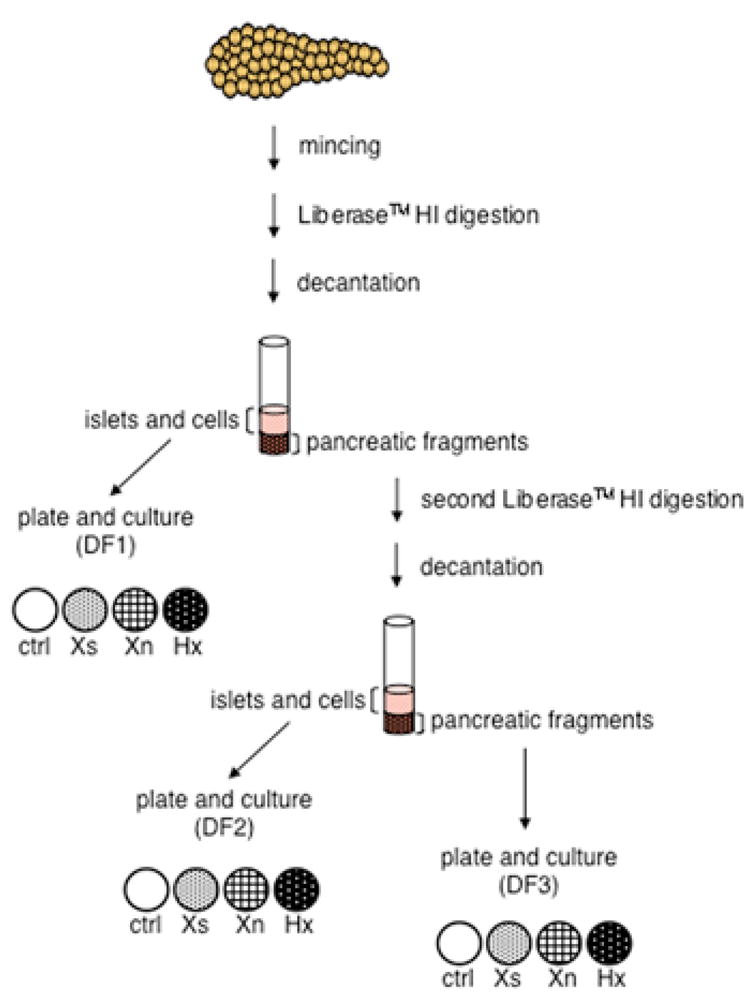
Schematic representation of the derivation of human pancreatic distributed stem cells Donor human cadaveric pancreas fragments were minced and serially digested. Released cells and islets were contained in two consecutive supernatant fractions (DF1 and DF2). A third fraction (DF3) was composed largely of residual tissue fragments. Material from all three fractions was cultured under 4 different conditions. Ctrl, no SACK agent supplementation; or supplementation with one of each of the three SACK agents, Xs, Xn, and Hx.

A 1 gram specimen from a normal pancreas of a 58 year old female donor was minced into approximately 1 mm3 pieces that were suspended in 10 mL CMRL-1066 medium (Invitrogen) containing 1 mg/mL Liberase HITM (Roche) and digested at 37°C for 20 minutes with agitation. Following digestion, tissue fragments and cells were sedimented by gravity for 30 seconds, at which point the supernatant was transferred to a fresh tube (DF1 in Figure 1) and supplemented with 5 mL dialyzed fetal bovine serum (dFBS; Sigma); and the sedimented material was resuspended in 10 mL medium, and the Liberase HITM digestion repeated. After the second digestion and 30-second gravity sedimentation, the second supernatant was transferred to a fresh tube (DF2 in Figure 1); and the final sedimented material was resuspended in 10 mL CMRL-1066 (DF3 in Figure 1). Five mL of dFBS was added to each tube. The three cell and tissue fractions were pelleted by centrifugation at 500g for 5 minutes and washed 3 times in 20 mL phosphate buffered saline (PBS; Invitrogen). The washed pellets were each resuspended in 8 mL CMRL-1066 medium containing 10% dFBS, 100U/mL penicillin, 100 μg/mL streptomycin, and 2 mM L-glutamine (Invitrogen). This mixture constituted the basic culture medium. The resuspended cells and tissue fragments were then equally divided in four 10-cm dia. culture dishes containing basic culture medium alone or basic culture medium supplemented to 1 mM concentration with one of three compounds (xanthosine, xanthine, or hypoxanthine; Sigma). Each culture and its passaged derivatives were maintained continuously in their initial SACK culture medium, which was refreshed every three days. A solution of 0.05% trypsin-EDTA (Invitrogen) was used to release adherent cells for passaging.

Cell colonies visible to the unaided eye after four weeks of culture were transferred individually into 1.56-cm dia. dish wells. When cultures reach 50% confluency, they were successively transferred into 3.5-cm dia. dish wells, 6-cm dia. dish wells, and 10-cm dia. culture dishes to expand. Cell colonies were counted after staining with crystal violet [26].

### Time-lapse microscopy

On the day preceding the start of the time-lapse recordings, examined cells were plated at a density of 1 × 10^4^ cells per well of a 6-well plate (3.5-cm dia. wells) in their maintenance culture medium. Twenty hours later, the culture medium was replaced with either SACK agent-free medium or medium supplemented with the respective SACK agent. After a 3 hour equilibration period in the standard incubator, plates were transferred to an environmental chamber for time-lapse microscopy imaging.

Parallel time-lapse microscopy was performed using an Observer. Z1 system (Zeiss). For each examination, a 40× magnification microscope field was imaged every five minutes for 96 hours using the Axiovision software. Movies were generated from each series of images and used to develop cell division pedigrees.

### Indirect *in situ* immunofluorescence (ISIF) analyses

Cells were placed on glass slides and fixed with 4% formaldehyde in PBS at room temperature for 20 minutes. Permeabilization was performed at room temperature for 10 minutes in 2% bovine serum albumin (Sigma), 0.2% dried milk, and 0.4% Triton X-100 (Sigma) in PBS. Blocking was done at 4°C for one hour in a 3% PBS dilution of the serum from the source-animal species of the secondary antibody. The primary antibodies were incubated overnight at 4°C with the cells after being diluted in their respective blocking buffer in the following ratios: rabbit polyclonal anti-Ngn3 (Chemicon) at 1:200; rabbit polyclonal anti-Glut2 (SantaCruz Biotechnologies) at 1:50; goat polyclonal anti-vimentin (Sigma) at 1:400; rabbit polyclonal anti-insulin and mouse monoclonal anti-glucagon (SantaCruz Biotechnologies) at 1:25; mouse monoclonal anti-Cpeptide (Millipore) at 1:25. Incubation with the secondary antibodies was also performed overnight at 4°C at the following dilutions in respective blocking buffers: goat anti-rabbit-FITC and donkey anti-goat-rhodamine (SantaCruz Biotechnologies) at 1:200; rabbit anti-mouse-AF568 (Invitrogen) at 1:400; rabbit anti-mouse FITC (Dako) at 1:200. The same procedures were used for ISIF with cryo-sections of differentiated cell clusters, except that the permeabilization step was extended to 30 minutes. Primary antibodies were titrated to optimize specific binding; and ISIF analyses exhibited no significant fluorescence when primary antibodies were omitted.

### Differentiation assays

Cells were induced to undergo pancreatic islet differentiation in SACK agent-free medium as previously described [27,28]. First, after trypsin treatment to release adherent cultured cells, the viable cell number was determined using a Vi-Cell XR Cell Viability Analyzer (Beckman Coulter). Approximately 5 × 10^5^ viable cells were transferred to a single well of a 6-well ultralow attachment plate (Costar) in CMRL-1066 supplemented with 100 U/mL penicillin, 100 μg/mL streptomycin, 1% fatty acid-free bovine serum albumin (Sigma), 2 mM L-glutamine, and 1X insulin-transferrin-selenium A (Invitrogen). Culture medium was refreshed daily for 4 days, when differentiated cell clusters were formed.

### Differentiated cell cluster cryo-section preparations

Suspended differentiated cell clusters were transferred to a conical tube and sedimented by gravity for 5 minutes. The culture medium was gently aspirated to leave approximately 100 μL of medium with the sedimented cell clusters. This concentrated cell cluster suspension was transferred to a 15 mm × 15 mm × 5 mm plastic tissue mold, covered with OCT™ (Tissue Tek), and flash-frozen on dry ice. Ten μm thick sections were generated using a cryostat (Leica CM3050 S) and immediately processed for ISIF.

### Cell injections in animals

Evaluated cells (1 × 10^6^) were each injected intraperitoneally into 6-week old *Nude-Foxn1nu* female mice (Harlan Laboratories). Injected animals were closely monitored for six months for signs of ascites development and tumor formation, including endpoint euthanization and autopsy. All mouse studies were approved by the Boston Biomedical Research Institute Animal Care and Use Committee to meet Office of Laboratory Animal Welfare guidelines.

## Results

### Adaption of the SACK method for expansion of clonal human adult pancreatic cell strains

We adapted the SACK method to expand clonal DSC strains from the adult human pancreas. Unlike previous pancreatic stem cell expansion studies that started with isolated islets [27,28], we started with unfractionated donor human pancreas by performing serial tissue enzyme digestions and culturing all cell fractions from the digestions (Figure 1). The resulting fractions of cells and tissue fragments were divided equally and cultured under four different conditions: medium without SACK agent supplementation (control) or media supplemented with one of three previously described SACK agents, *i.e.* xanthosine (Xs), xanthine (Xn), or hypoxanthine (Hx).

**Figure 2:**
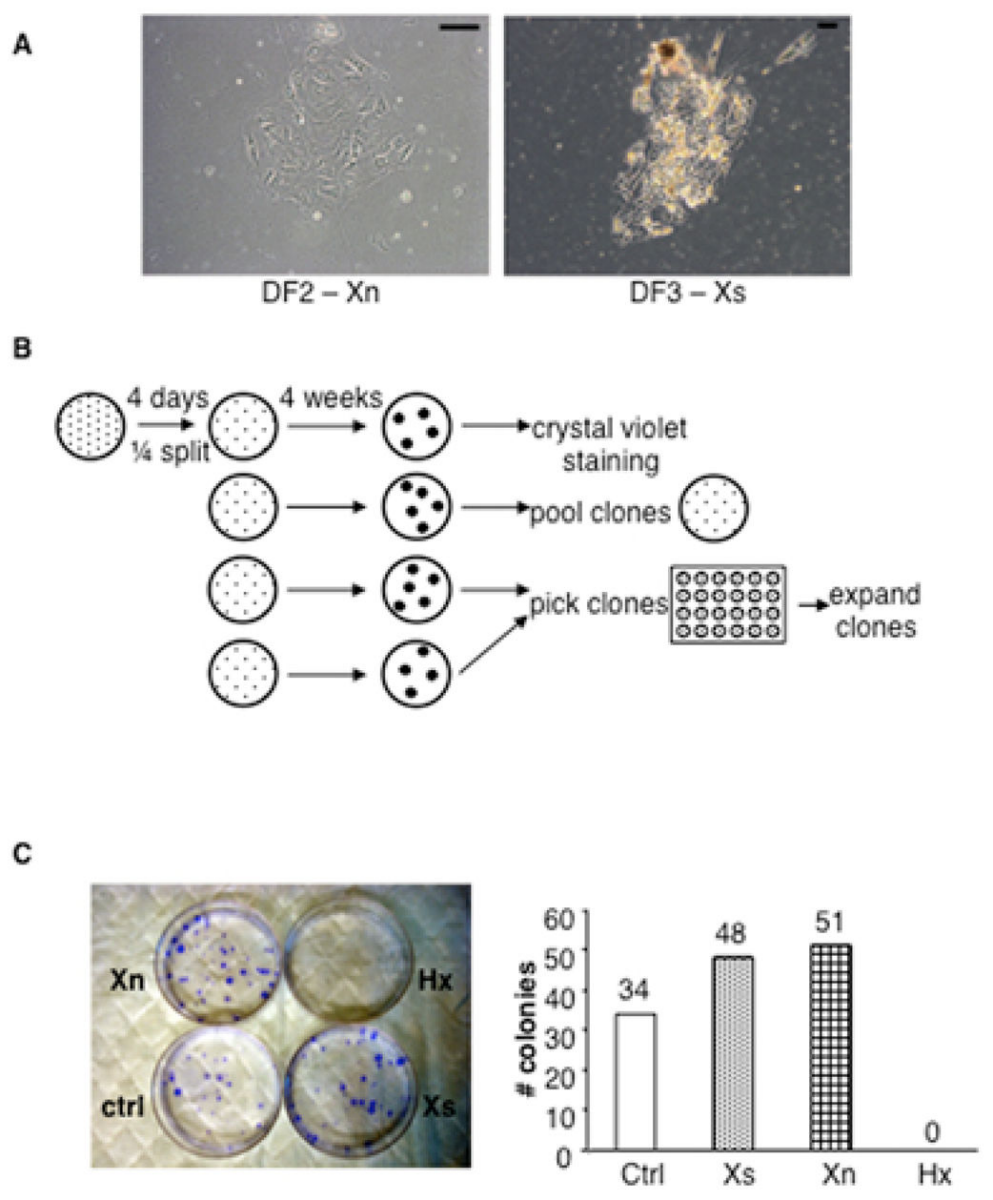
The SACK agents Xn and Xs increase the efficiency of human pancreatic cell strain expansion **A.** Phase micrograph examples of primary cell colonies obtained after 4 days of culture of pancreatic cells and fragments from fractions DF2 and DF3 (see Figure 1) in Xn supplemented and Xs-supplemented medium, respectively. Scale bars, 100 μm. **B.** Scheme for derivation of secondary colonies and cell strains. Each culture of primary colonies was trypsinized and divided equally among four new cultures in the respective culture medium. Four weeks later, replicate cultures were used to estimate SACK agent effects on secondary colony formation and derive pooled and clonal cell strains. C. SACK agent effects on secondary colony formation efficiency of DF3-derived cells. Left, examples of crystal violet-detected DF3 secondary colonies with respect to SACK-agent supplementation. Right, quantitative analysis of SACK-agent effects on the formation efficiency of DF3-derived secondary colonies.

After an initial four days in culture, primary cell colonies were observed under all conditions for all fractions (examples in Figure 2A). No significant differences were noted in the distribution of colony morphologies obtained among the different conditions and fractions. At this point, each culture of primary colonies was trypsinized as a cell pool and equally sub-divided to make four replicate cultures that maintained the same SACK condition as the parent culture (Figure 2B). The new cultures were grown for four weeks, at which time secondary cell colonies became visible to the unaided eye. However, only cultures derived from the final digestion fraction (Figure 1, DF3) produced secondary colonies. Supplementation with the SACK agents Xs or Xn increased the yield of secondary cell clones by as much as 50% (Figure 2C). In contrast, no secondary colonies were produced under conditions of Hx supplementation.

### Asymmetric cell kinetics properties of SACK-expanded human adult pancreatic cell strains

**Figure 3:**
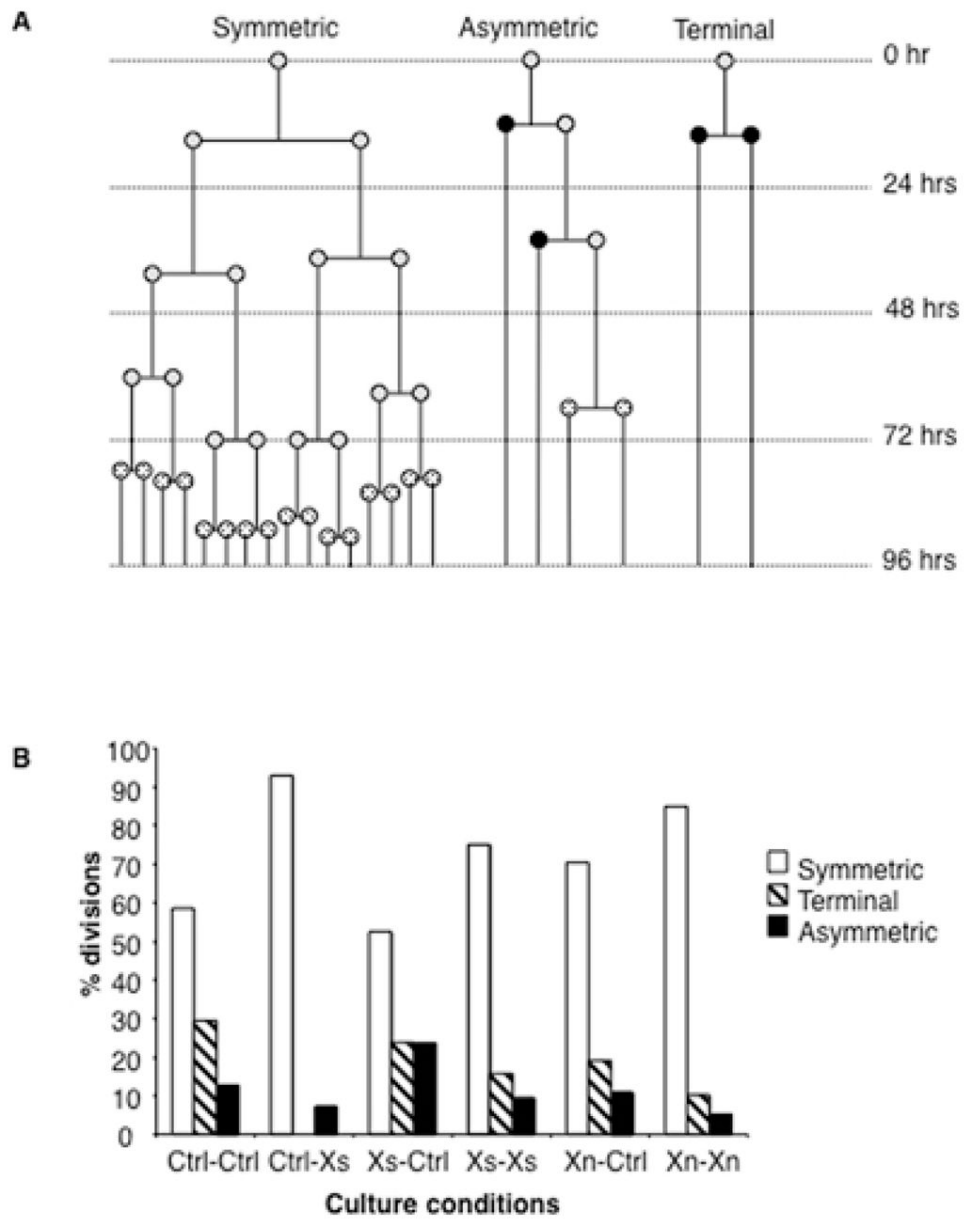
SACK-expanded human pancreatic cell strains exhibit SACK-agent suppressible asymmetric cell kinetics **A.** Examples of time-lapse determined cell division pedigrees defined as Symmetric, Asymmetric, and Terminal. Shaded circle, dividing cell; black circle, nondividing cell; stippled circle, undetermined division fate. **B.** Quantitative analyses of percent changes in the representation of cell kinetics division types shown in A with respect to either SACK-free conditions (-Ctrl) or respective SACK-agent supplementation (-Xs, -Xn). Data from experiments with control (Ctrl-), Xs-expanded (Xs-), and Xn-expanded (Xn-) HuPan cell strains are shown. The respective numbers of informative divisions included were 24, 28, 21, 32, 37, and 59. p < 0.001 for the overall shift to symmetric cell kinetics by Fisher’s two-tailed exact test.

For an initial evaluation of the cell kinetics properties of expanded cells, we performed time-lapse microscopy analyses of pooled cultures of secondary colonies (Figure 2B, pooled clones). We evaluated cells from the pooled cell strains derived from the DF3 digestion fractions using nonsupplemented, Xs-supplemented, and Xn-supplemented media. Digital time-lapse phase microscopy imaging was performed for 96 hours, which corresponds to approximately 4 cell generations. The imaging data were used to develop cell division pedigrees for each type of pooled cell strain under control conditions (*i.e.,* without SACK agent supplementation), or under conditions of supplementation with the respective SACK agent used for their expansion. Xs was used for the control strains, which were expanded without SACK agent supplementation. As predicted for SACK, the mean cell cycle time for dividing cells did not vary significantly for the three comparisons of SACK-free *versus* SACK supplementation conditions (mean difference = 7±5%); and no significant difference was noted in the initial fraction of non-dividing cells in either condition. However, for the three strain types overall, supplementation with a SACK agent shifted their cell division patterns to increased symmetric cell kinetics, as predicted for SACK based cell expansion (Figure 3B; p < 0.001; Fisher’s two-tailed exact test).

The SACK agent-induced cell kinetics shift was evident from changes in the relative frequencies of three defined cell division patterns: symmetric, asymmetric, and terminal [11,29,30] (Figure 3A for examples). Symmetric divisions produced two cells that divided during the time-lapse imaging period. Asymmetric divisions produced two cells, one that divided and one that did not during a period of imaging equal to at least twice the mean cell cycle time. Terminal divisions produced two cells, but neither divided thereafter, based on the same imaging period requirement used for asymmetric divisions. Terminal divisions are also indicative of overall asymmetric cell kinetics [11]. SACK-agent supplementation resulted in an increase in the frequency of symmetric divisions with a corresponding decrease in the frequency of cells undergoing asymmetric and terminal divisions (Figure 3B). The time-lapse analyses confirmed that supplementation with SACK agents suppressed the asymmetric cell kinetics of some tissue cells, which in this specific case were predicted to be adult human pancreatic DSCs.

### Clonal SACK-expanded human adult pancreatic cell strains have properties of pancreatic DSCs

Clonal derivation is required for establishing the degree of differentiation potency of expanded cell clones. Isolated, individual secondary colonies were evaluated for clonability in their respective expansion media (Figure 2B). Control secondary colonies expanded in SACK agent-free medium had a successful rate of clone expansion of 3.8%, corresponding to only 1 successful colony expansion out of 26 trials. In contrast, for the more abundant Xs-derived and Xn-derived secondary colonies, the average clone expansion success rate was 12%, resulting in 6 and 7 expanded cell clones, respectively. The higher cloning success rate is consistent with higher rates of symmetric cell kinetics.

**Figure 4:**
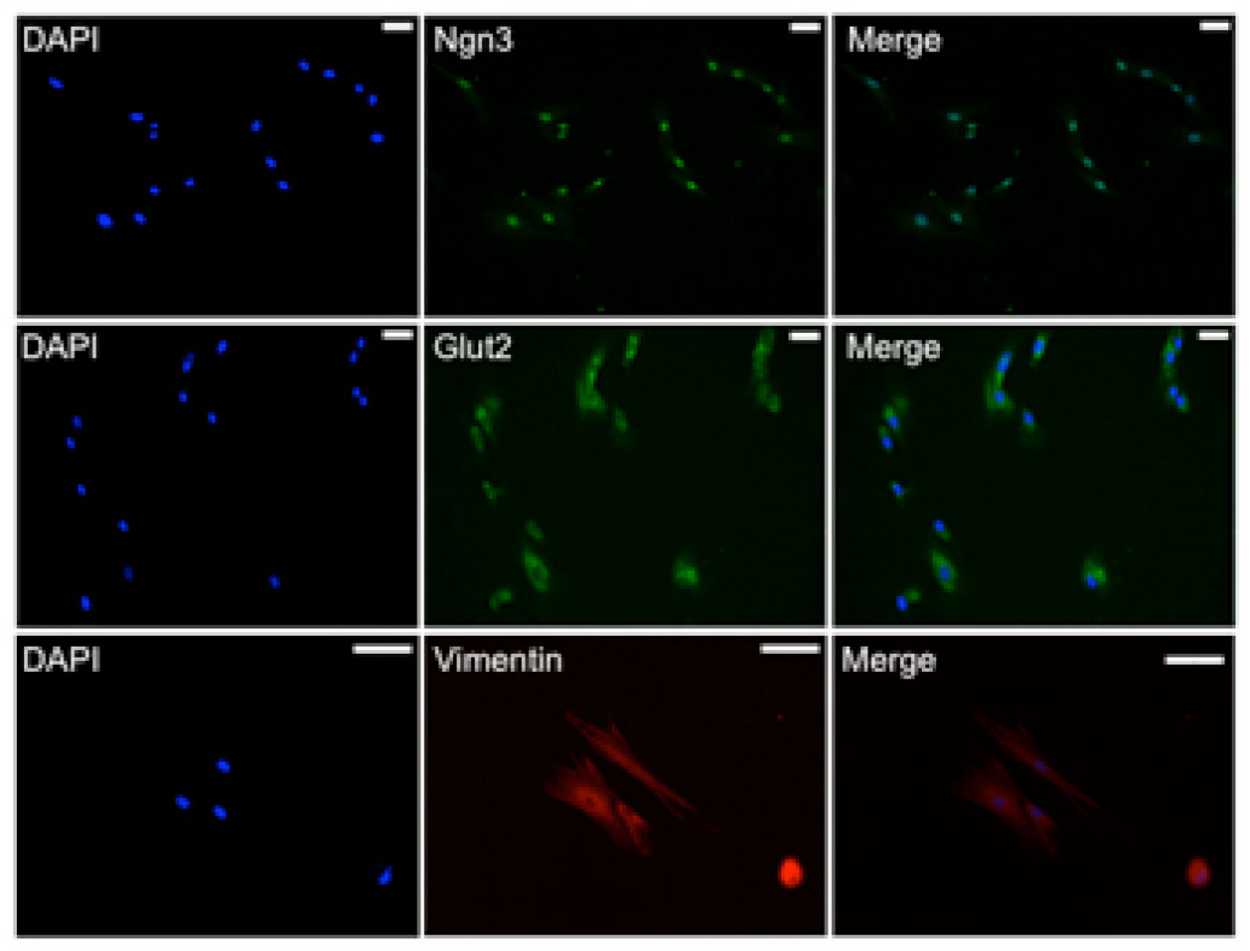
SACK-expanded HuPan DSC strains co-express pancreatic endocrine and mesenchymal markers under conditions of active proliferation Example of ISIF micrographs for HuPan DSCs showing positive nuclear staining for the endocrine progenitor transcription factor Ngn3 (top panels) and positive cytoplasmic staining for the pancreatic lineage marker Glut2 (middle panels) and the mesenchymal cell marker vimentin (bottom panels). DAPI, nuclear DNA epifluorescence. Scale bars, 50 μm.

The observed SACK agent-dependent cell kinetics pattern shifts and increased cloning success rate are identifying properties of DSCs [17]. To establish that the expanded DSC strains had specific potency for pancreatic developmental lineages, we evaluated them by indirect *in situ* immunofluorescence (ISIF) for expression of pancreas-specific molecular markers under conditions for expansion. As related below, when these markers were detected, cells uniformly expressed them at a frequency > 95%. Of the tested transcription factors, Pdx1, Ptf1a, Ngn3, and Arx, only the embryonic endocrine precursor marker neurogenin-3 (Ngn3) [31] was detected in SACK-expanded “HuPan” cells (Figure 4, top row). However, the cells also expressed glucose transporter-2 (Glut2; Figure 4, middle row), which is a characteristic of pancreatic endocrine cells involved in glucose metabolism. We also found that HuPan strains showed strong cytoplasmic expression of vimentin, a mesenchymal cell marker (Figure 4, lower row), consistent with the mesenchymal morphology of HuPan cells.

**Figure 5:**
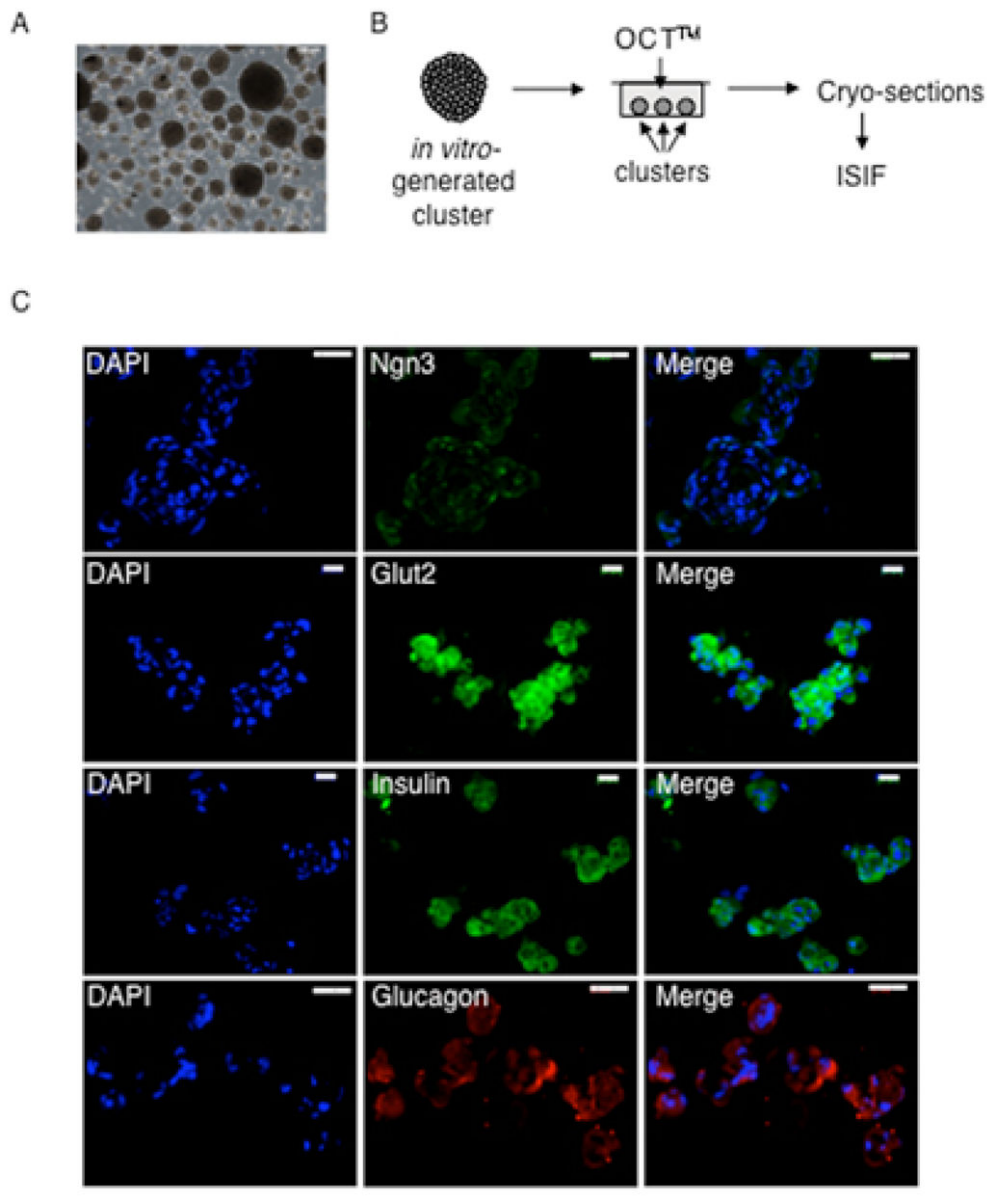
SACK-expanded HuPan DSC strains produce differentiated cells that express insulin and glucagon **A.** Phase micrograph showing examples of cell clusters produced after 4 days of culture of HuPan cell strains under conditions that promote pancreatic lineage differentiation. Scale bar, 100 μm. **B.** Scheme for characterization of cell lineage phenotypes in differentiated cell clusters by ISIF (See *Materials and Methods*). **C.** Representative epifluorescence micrographs of HuPan cells after ISIF with specific anti-Ngn3, anti-Glut2, antiinsulin, and anti-glucagon antibodies as indicated. DAPI, nuclear DNA epifluorescence. Scale bars, 50 μm.

There are many previous reports of isolation of adult pancreas-derived mesenchymal cells that can differentiate into insulin-producing cells [27,28,32]. However, to our knowledge, none of these express Ngn3 in their normal state. To evaluate whether expanded HuPan cultures contained pancreatic DSCs possessing the ability to produce differentiated progeny cells with properties of mature pancreatic cells, we performed *in vitro* differentiation experiments using culture conditions previously established for the formation of islet-like cell clusters in suspension [27,28]. All evaluated HuPan strains formed islet-like cell clusters of similar size with similar efficiency (Figure 5A). These included pooled and clonal Xs-derived and Xn-derived strains, and pooled control HuPan strains. To determine their degree and type of differentiation, we performed ISIF analyses for specific pancreatic endocrine differentiation markers with cryosections from frozen islet-like cell clusters (Figure 5B). In differentiation analyses with the pooled HuPan strains, the endocrine progenitor cell marker Ngn3 was still expressed, showing strong focal expression in nuclei (Figure 5C, top row). Strong cytoplasmic expression of Glut2 was also detected, as well as cytoplasmic expression of both insulin and glucagon (Figure 5C, middle and bottom rows, respectively). Apparent co-expression of insulin and glucagon, combined with the persistence of Ngn3 expression, suggested that under the *in vitro* conditions used, HuPan cells produced differentiated progeny cells that matured to a new precursor cell state with potential for production of cells with mature α-cell or β-cell function.

**Figure 6:**
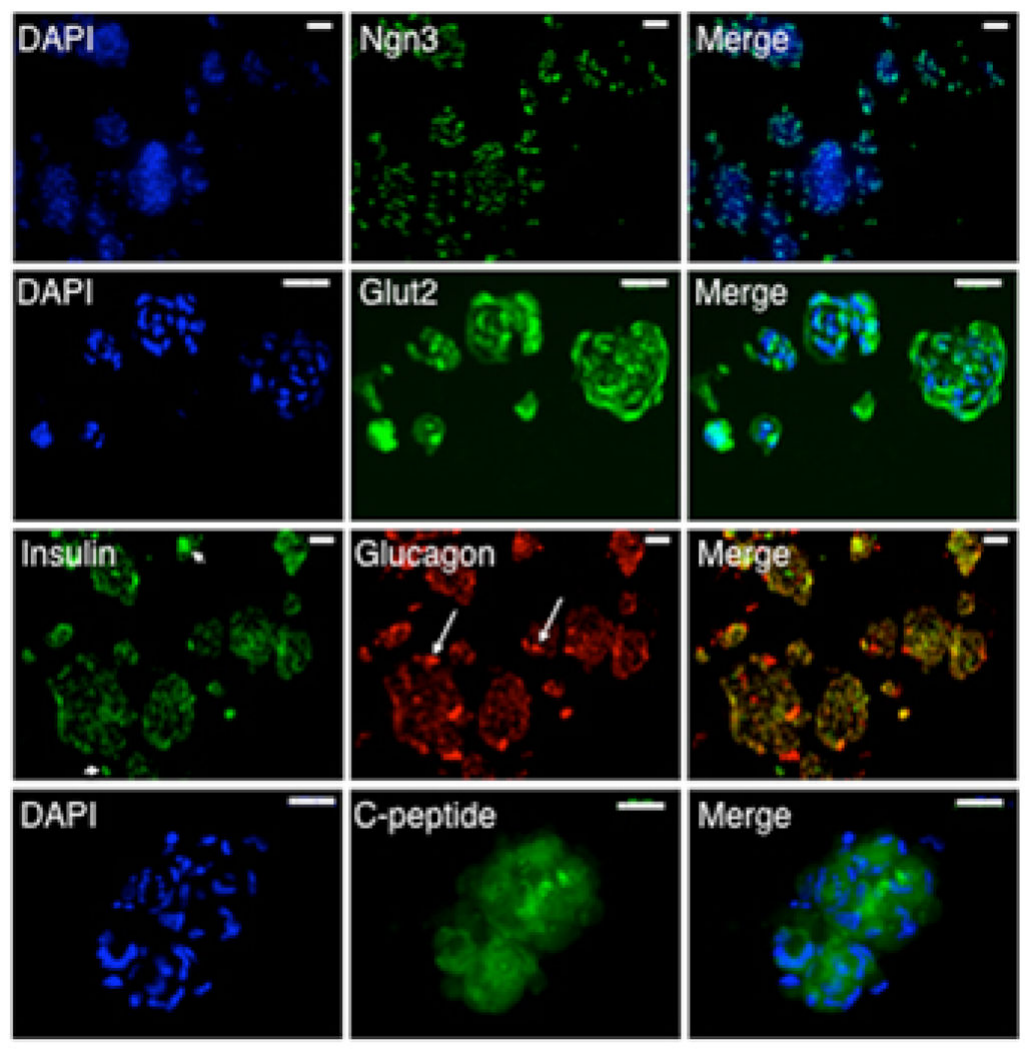
Differentiated progeny cells produced by clonal HuPan DSCs co-express insulin and glucagon Shown are examples of epifluorescence micrographs of ISIF analyses of cryosections of differentiated cell clusters produced from a clonal HuPan DSC strain. Top row, anti-Ngn3. Second row, anti-Glut2. Third row, dual ISIF with anti-insulin and anti-glucagon antibodies. Arrowheads, indicate examples of cells expressing only insulin. Arrows indicate examples of cells producing only glucagon. Bottom row, anti-C-peptide. DAPI, nuclear DNA epifluorescence. Scale bars, 50 μm.

Differentiation studies with clonal HuPan cells confirmed production of multi-potent cell lineages from single precursor cells. Again, we observed the persistence of Ngn3 and Glut2 expression in the clusters of differentiated progeny cells, as well as co-expression of both glucagon and insulin (Figure 6). Moreover, we confirmed co-expression of insulin and glucagon by determination of direct epifluorescence overlap in dual-antibody ISIF experiments (Figure 6, insulin-glucagon merge). Finally, we also detected cytoplasmic proinsulin C-peptide, which is present in insulin precursor proteins. Its detection confirmed that *de novo* insulin synthesis occurred in the examined cells. These analyses showed that the clonally expanded HuPan DSCs could produce differentiating progeny cells with the potential to produce both α- and β-cell lineages.

Though infrequent, we also found examples of differentiated cells that expressed only insulin (Figure 6, arrowheads) or only glucagon (Figure 6, arrows) in differentiated cell clusters. These may be more mature, fully specified cells. Observing these cells indicates that the SACK-expanded HuPan cells can also produce cells with potential to mature into functional α- and β-cells.

### SACK-expanded HuPan pancreatic stem cells are non-tumorigenic in mice

To evaluate the tumor-forming potential of HuPan cells, we injected 1 × 10exp6 control, Xs-derived, and Xn-derived pooled HuPan cells into the peritoneal cavity of two 6-week old female *Nude-Foxn1nu* mice. After a six month examination period, no signs of ascites or tumor masses were detected in any of the injected mice. Autopsies were performed at that point, and no evidence of tumor was found for all tissues examined.

## Discussion

The SACK method was previously shown to be effective for expansion of DSCs from several different mammalian tissues of diverse mammalian species [14,16,17,20–23]. Here, we report a first adaption and evaluation of the method for expansion of adult human pancreatic DSCs. The new method increased the efficiency of expansion of pooled and clonal pancreatic DSC strains from normal adult human donors. The novel cell strains, called “HuPan” cells, express molecular markers of pancreatic endocrine identity and glucose metabolism and show both α-cell and β-cell differentiation potency.

Based on this initial success, we consider that in the future the SACK method may provide further advances for the production of human pancreatic DSCs of sufficient quantity and quality to enable their evaluation for T1D transplantation therapy. To date, our highest degree of expansion with HuPan strains was 20 population doublings. However, this degree of expansion was only possible with a pooled DSC strain; and clonal HuPan strains exhibited more limited expansion (data not shown). Though 20 population doublings can provide adequate cells for future evaluations in mouse transplantation models for T1D, more extensive and reliable expansion will be required to support human clinical trials. Therefore, our future studies will focus first on better optimization of SACK agent supplementation and the integration of other *in vitro* factors (*e.g*., pancreatic extracellular matrix) that might further extend this early expansion success.

The SACK method is based on the principle of using rGNP salvage precursors to convert asymmetrically self-renewing DSCs to symmetric self-renewal, which promotes their selective exponential expansion. Because the conversion is reversible, when SACK agents are removed *in vitro* or *in vivo*, the expanded stem cells can regain their full tissue renewal function [14,15,20].

Compared to other previously described methods for establishment of adult human pancreatic stem cell lines, another significant difference that we adapted was the use of intact pancreas as starting material instead of only isolated islets. If pancreatic DSCs are not tightly associated with islets, this strategy will avoid their inadvertent loss. Extensive re-digestion of pancreatic tissue also appeared important for successful expansion. Whether this feature reflects properties of the location of pancreatic stem cells in the tissue architecture is presently uncertain. However, based on studies of mouse Ngn3^+^ progenitor cells induced in a mouse pancreatic injury model, there is a suggestion that these cells might reside among cells that line the pancreatic ducts [33].

The new approach yielded adult human cell strains with properties indicative of pancreatic endocrine DSCs. Consistent with the theory for their derivation, the expanded HuPan DSC strains exhibit asymmetric self-renewal kinetics that shifts to symmetric self-renewal kinetics in response to the rGNP precursors, Xn and Xs, which were the SACK agents used to derive the cells. A rare control cell strain, which expanded without purine supplementation, also shows SACK-agent induced shifts to greater symmetric self-renewal. The pancreatic lineage status of this cell strain was found to be the same as the ones derived with SACK agents (data not shown). Its derivation supports the idea that the SACK method only increases the efficiency of expansion of pancreatic DSCs, and does not otherwise alter the cells.

Both pooled and cloned HuPan cell strains have the ability to produce numerous bipotential precursor cells that display phenotypes attributed to both α-cell and β-cell lineages. Under conditions of SACK-supplementation, HuPan cell cultures show expression of the embryonic endocrine precursor marker Ngn3 and the pancreatic lineage marker Glut-2. However, cells expressing the mature differentiation markers, insulin and glucagon, are not detected under these culture conditions. Under SACK-free conditions that foster pancreatic cell differentiation, many new cells appear that retain expression of Ngn3 and Glut-2, but now also express insulin and glucagon. These features distinguish HuPan cells from previously described expanded adult human pancreatic cells.

The co-expression that we observe is consistent with a differentiated intermediate precursor descended from undifferentiated HuPan cells. It is noteworthy that about 5% of cells in midgestational pancreatic anlage also express both insulin and glucagon. These cells are present when fetal β-cell and α-cell expansion is occurring. However, they are not observed in neonatal human pancreas, when pancreatic growth ebbs. These multi-potent fetal pancreatic cells also do not appear to cycle [34–36]. This feature is the basis for the suggestion that they are an intermediate stage before full commitment to specific single-hormone secreting cell types, like β-cells. We have not yet evaluated the cell cycle properties of the multi-potent cells produced in differentiated HuPan cultures, but these future studies could be informative regarding their function in the production of mature pancreatic cell types.

Though most of the differentiated cells found in the *in vitro*-generated islet-like cell clusters express both insulin and glucagon, a sub-population of cells also shows exclusive expression of only one of the markers. Although a precursor-product relationship is not yet confirmed, the presence of these single-marker cells supports the idea that the more abundant bipotent cells are committed precursors that produce mature α-cells and β-cells inefficiently *in vitro*. These properties are encouraging that, after return to an appropriate *in vivo* environment, HuPan cells may effectively reconstitute deficient α-cell and β-cell function.

At this stage of our characterizations, we have not limited HuPan cells only to α-cell and β-cell potency. In previous SACK studies with rat liver, both hepatocytic stem cell strains and cholangiocytic stem cells strains arose from the same SACK expansion trials [14]. This result can occur because the only essential requirement for SACK success is an efficient reversible shift in stem cell self-renewal state from asymmetric to symmetric. Therefore, given that the *ex vivo* environment is permissible for them to survive and proliferate, different types of DSCs can emerge simultaneously from the same tissue preparation, especially after clonal derivation. So, some HuPan clones could be DSCs that renew pancreatic δ-cells, ε-cells, or PP-cells. Moreover, multi-potent HuPan strains may emerge that can give rise to all classes of mature pancreatic cells. In the mouse, there is evidence that Ngn3^+^ cells induced by injury can produce α-, β-, δ-, and PPcells [33].

The prime clinical indication for production of pancreatic DSCs is their potential to restore insulin-secreting β-cells after transplantation for the treatment of T1D. Our differentiation studies show that HuPan DSCs have bipotency for α-cells and β-cells. Even though restoration of mature glucose-responsive β-cells is the mainstay for T1D treatment, the interaction between α-cells and β-cells is also important for optimal regulation of glucose utilization by mature islets [37]. Despite continuing innovation in blood glucose monitoring for insulin replacement therapy for T1D, suboptimal regulation of blood glucose levels persists as the cause of the chronic debilitating complications (*e.g.* retinopathies, nephropathies, heart disease) faced by T1D patients. The availability of stem cells able to restore and renew both α-cells and β-cells simultaneously would offer the possibility of reducing the morbidity of T1D even further after successful cell transplantation. The successful SACK-expansion of HuPan DSCs described in this report establishes this potential.

## Abbreviations

DF: Digestion Fraction
dFBS: Dialyzed Fetal Bovine Sérum
DSC: Distributed Stem Cell
Glut2: Glucose Transporter-2
hESC: Human Embryonic Stem Cell
HSC: Hematopoietic Stem Cell
Hx: Hypoxanthine
IMPDH II: Type II Inosine Monophosphate Dehydrogenase
iPSC: Induced Pluripotent Stem Cell
ISIF: Indirect *in situ* Immunofluorescence
Ngn3: Neurogenin-3
PBS: Phosphate-buffered Saline
rGNP: Guanine Ribonucleotide
SACK: Suppression of Asymmetric Cell Kinetics
T1D: Type 1 Diabetes
Xn: Xanthine
Xs: Xanthosine
